# Challenges in the use of the mental health information system in a resource-limited setting: lessons from Ghana

**DOI:** 10.1186/s12913-018-2887-2

**Published:** 2018-02-08

**Authors:** Lily Kpobi, Leslie Swartz, Angela L. Ofori-Atta

**Affiliations:** 1 Department of Psychology, Alan J. Flisher Centre for Public Mental Health, Stellenbosch University, Private bag X1, Matieland, Stellenbosch, 7600 South Africa; 2 Department of Psychiatry, University of Ghana School of medicine and Dentistry, Korle Bu, Accra, Ghana

**Keywords:** Ghana, Mental health information systems, Implementation, Challenges

## Abstract

**Background:**

One of the most successful modes of record-keeping and data collection is the use of health management information systems, where patient information and management plans are uniformly entered into a database to streamline the information and for ease of further patient management. For mental healthcare, a Mental Health Information System (MHIS) has been found most successful since a properly established and operational MHIS is helpful for developing equitable and appropriate mental health care systems. Until 2010, the system of keeping patient records and information in the Accra Psychiatric Hospital of Ghana was old and outdated. In light of this and other factors, a complete reforming of the mental health information systems in three psychiatric hospitals in Ghana was undertaken in 2010. Four years after its implementation, we explored user experiences with the new system, and report here the challenges that were identified with use of the new MHIS.

**Methods:**

Individual semi-structured interviews were conducted with nine clinical and administrative staff of the Accra Psychiatric Hospital to examine their experiences with the new MHIS. Participants in the study were in three categories: clinical staff, administrator, and records clerk. Participants’ knowledge of the system and its use, as well as the challenges they had experienced in its use were explored using an interpretative phenomenological approach.

**Results:**

The data suggest that optimal use of the current MHIS had faced significant implementation challenges in a number of areas. Central challenges reported by users included increased workload, poor staff involvement and training, and absence of logistic support to keep the system running.

**Conclusions:**

Setting up a new system does not guarantee its success. As important as it is to have a mental health information system, its usefulness is largely dependent on proper implementation and maintenance. Further, the system can facilitate policy transformation only when the place of mental health in district, regional and national health discourse improves.

## Background

The WHO estimated in 2011 that 28% of countries had no specific budget for mental health; and further, for the countries that did have a budget, an estimated 37% spent less than 1% of their health budget on mental healthcare and services [1]. For many low and middle-income countries (LMICs), budgetary allocation for mental health is often low and remains a significant problem in ensuring positive health outcomes. In Ghana, for instance, some authors have argued that, of the over two million individuals reported to be suffering from mental illness, 95% of these do not have access to formal mental health services [1, 2].

Information on specific disorders, their prevalence and their outcomes within specific contexts is key in policymaking and planning, in order to understand the mental health situation in any country. One of the most successful ways of obtaining such information is through the use of health management information systems, where patient information and care plans are uniformly entered into a database for ease of further patient management. For mental healthcare, a mental health information system (MHIS) has been described as most useful [3].

According to the WHO, a mental health information system (MHIS) is “a system for collecting, processing, analysing, disseminating and using information about a mental health service and the mental health needs of the population it serves” (p. 13) [3]. It can help to develop equitable and appropriate care systems within a specific context [4], and can help to improve clinician communication, adherence to best-practice norms and reducing errors in clinical care [4]. The MHIS therefore affects planning, budgeting and evaluation at all levels of health care. It is also useful for presenting mental health data in an understandable and accessible form to stakeholders – including service providers, service users, policy-makers, and the general population [3].

A well-functioning MHIS is an important factor for mental healthcare systems in any country [5, 6]. With an operational system in place, it provides concrete data to policy-makers and planners, and allows for targeted interventions and evaluations to be developed [7]. In addition, mental healthcare providers are able to identify patterns in presenting complaints and medication needs of their populace, which in turn informs service delivery and practice. Thus, an MHIS facilitates the provision of evidence-based services and can contribute to equity and accuracy of care. For the general population, information obtained from the system is a good way of knowing what kinds of services are available to them [3].

A mental health information system is therefore a useful tool for patient management and monitoring. It allows information to be kept in a centralized, uniform manner which is valuable for long-term care [8], particularly when clinicians or healthcare facilities change. Such data can be easier to access whenever further treatment is required. It can also be used to generate epidemiological reports, thereby creating a better picture of the mental health situation in a hospital, district or country.

Even though the MHIS has obvious benefits, its usefulness may be compromised by various factors. These factors include problems in the initial design of the information framework [8] which should ideally make the system easy to access and use by the relevant stakeholders. To achieve this ease, identifying and incorporating relevant indicators and categories of data to be collected is key.

Apart from design problems, further problems may arise during implementation of the system [3]. One of the foremost problems in the implementation of MHIS in LMICs is inadequate resources [9]. A good MHIS requires investment in staff who need to be properly trained in how to collect, process and analyse the data. In addition, data that are collected through the system should be reported in a manner which is meaningful and accessible to potential or actual users, otherwise it defeats the purpose of facilitating equity and improved healthcare.

A fully electronic system further requires investment in the relevant hardware and software necessary to install and maintain the system [7, 9]. The user interface of this system must also be user-friendly and not necessarily require advanced technical knowledge to operate. Given the limited number of healthcare professionals in many LMICs, the MHIS should ideally be quick and easy-to-use. This can further help to ensure that the type and quality of data obtained is optimal [5, 7].

A number of studies have reported the process of implementing new MHISs in different countries, including the various challenges that were experienced in each of these [5, 10, 11]. Some of the challenges that have been identified in the implementation of MHIS in other LMICs include lack of policies to govern information management in mental health [12], insufficient or poorly trained personnel [13], as well as problems in the workflow processes and mechanisms [14]. Despite these challenges, opportunities for growth and successful implementation abound if lessons are learned from the experiences of other countries.

### Mental health information systems in Ghana

In their situation analysis of mental health care in Ghana, Ofori-Atta et al. [2] reported that there were inadequate policies, legislation and services for mental health in the country. They suggested a number of factors as accounting for these; one of the major problems identified was a weak mental health information system (MHIS). The system which existed at the time (in 2008) collected information on only 4 categories of mental illness (i.e. psychoses, neuroses, epilepsy, and substance use disorders) and together, made up less than 1% of the data collected by the Ghana Health Service. The situation analysis also revealed that these diagnoses were not standard and therefore could not provide credible information on the mental health needs and services in the country [2].

The systems of record keeping and management of patient data in the Accra Psychiatric Hospital (where the current study was situated) were therefore old and outdated. Patient data were largely kept in paper-based folders. These could potentially be misplaced or mishandled. In addition, care providers were reportedly using different diagnostic systems in managing patients [2], resulting in an absence of standardized information. Such discrepancies presented challenges for policy development and advocacy.

In light of this and other factors, a research programme consortium (called the Mental Health and Poverty Project, MHaPP) among other things designed a new, semi-computerized MHIS for the three psychiatric hospitals in Ghana in 2010. With this new system, data are recorded first on paper, and then entered into an online database by clerks. The MHaPP implemented this new MHIS by providing logistics, equipment and partial support to run the system. The intervention also included training of users such as physicians, nurses, clerks and physician assistants (see MHaPP final report) [15]. The aim of this intervention was to attempt to facilitate policy change and funding for mental health through the provision of reliable and credible data on mental health care needs and services in Ghana.

The three psychiatric hospitals in Ghana therefore received a more modern system to aid in patient management and care. Although much has been written on the implementation of the information systems, little is known about the current state of the system in Ghana since its implementation. The aim of this paper is therefore to discuss the experiences of users of the MHIS at the Accra Psychiatric Hospital, and the challenges that they encountered with this new system. We believe this is an important first step towards identifying areas of the system that require strengthening so that the MHIS achieves its intended purpose of improving mental healthcare and delivery in Ghana. The data reported here form part of a broader study of user experiences with the new MHIS but we focus here on the reported challenges (for more details of the larger study, see Kpobi) [16].

## Methods

### Research setting

The current study was conducted in the Accra Psychiatric Hospital, located in the capital city of Ghana, Accra. This hospital is the oldest and largest psychiatric care facility in the country [17]. Being the first of only three public psychiatric hospitals in Ghana, the Accra Psychiatric Hospital treats the largest number of psychiatric cases per year in the country [17]. Its management and staff were also reportedly key players in the implementation of the new MHIS. As a result, it was anticipated that staff of the hospital would have extensive experience in using the new system.

### Research participants

The research participants for this study were drawn from three different categories of staff at the hospital: clinical staff (including doctors, medical/physician assistants and nurses), records staff and administrative staff. Getting participants for this study was surprisingly difficult, despite the relatively small number of clinical staff at the hospital who had experience with the new system. Informal enquiries revealed a deep distrust of outsiders on the part of some of the staff as a result of an undercover news report that had been conducted at the hospital shortly before we began data collection [18]. Thirteen members of staff who were approached refused to participate, and those who did consent to participate were sometimes reluctant to be recorded. The staff who declined participation were primarily nurses and records staff.

At the time of data collection, there were five psychiatrists at the hospital, four residents-in-training, and six medical/physician assistants. The final sample of clinical staff that participated in the study consisted of two doctors (one specialist psychiatrist and one psychiatric resident), two medical/physician assistants, and three mental health nurses. In addition, one data entry clerk (who was the only one willing to participate) was interviewed out of the four employed by the hospital for data entry purposes. Lastly, the medical director was interviewed to explore administrative challenges that may be present with the new MHIS. The medical director readily agreed to be interviewed.

Thus, the total number of staff interviewed was nine. The doctors comprised one male and one female; the medical/physician assistants were all male; and the nurses were all female. The clerk and the administrator were both male. A summary of participant categories is presented in Table 1 below:

**Table 1:** Study participant codes and categories

Participant code	Staff category	Gender
M1	Medical assistant	Male
M2	Medical assistant	Male
D1	Doctor/Psychiatrist	Male
D2	Doctor/Psychiatrist	Female
N1	Mental health nurse	Female
N2	Mental health nurse	Female
N3	Mental health nurse	Female
C1	Data entry clerk	Male
A1	Medical director	Male

### Procedure

Institutional ethics approval for this study was obtained from Stellenbosch University and the Ghana Health Service. In addition, permission was sought from the Accra Psychiatric Hospital. Following institutional permission, individual written informed consent was obtained from each participant.

As stated above, the data reported here form part of a broader study of the MHIS at the Accra Psychiatric Hospital. The first phase of that broader study involved an audit of archived records of the MHIS (more in-depth explanation of this phase is available) [16]. From the archival audit, potential staff who had experience in using the MHIS were identified. This paper reports on phase two of the study which involved individual, semi-structured interviews with nine of the identified clinical and administrative staff.

Potential participants were approached, and the objectives of the study were explained to them. As indicated above, not all the clinical staff who were approached were willing to participate. Those who gave consent to participate were interviewed. The medical director however, did not hesitate to take part in the study.

All interviews were conducted in English and were conducted by the first author. Interviews were recorded with a tape recorder when written consent to record was provided. Three of the participants (two nurses and one medical assistant), asked not to be recorded; their responses were therefore written down. Interviews were conducted in the consulting rooms of the various clinical staff, and the private office of the administrator. The average time for the interviews was 35 min.

Participants were asked a range of questions about their work with the MHIS including questions such as “How often do you use the new system?”, and “Has the new system affected your work in any way?” Participant validation processes were carried out, though it is important to note that most of them declined to participate in this.

### Data analyses

All recorded interviews were transcribed verbatim. Interviews which were not recorded had hand-written notes and responses which were typed and coded. The data were inductively analysed using Interpretative Phenomenological Analyses (IPA) with the help of ATLAS.ti software (v.6). First, all transcripts were thoroughly read several times to explore for initial meaningful patterns and trends. Each interview was then coded, and the codes were grouped, based on the emerging ideas that were expressed by the participants. In particular, we focused on the meanings they had attached to their experiences with the new system. These explanations have been organized into themes and are discussed as results below.

## Results

Various challenges were identified from staff experiences with the new MHIS at the Accra Psychiatric Hospital. These include increase in the staff workload; cumbersome data capture process; perceived limited staff involvement in the implementation process; poor resource allocation; and dissemination of data. Each of these themes is discussed in more detail below.

### Increased staff workload

The foremost challenge identified by our participants was the burdensome nature of the new MHIS for staff. The new system is partially electronic with patient information being collected first on paper through forms which are filled out by nurses and doctors, and later being entered into an electronic database [15, 16]. This semi-computerized system was put in place due to the limited number of computers available at the hospital. Six of the nine participants complained that the hybrid system duplicated work and this is particularly problematic where clinical staff resources are scarce. One medical assistant put it this way:

“*…now the work is more because…the time we spent on one patient is more…we have to fill out the information on the form after each patient…this takes time away from what we could have spent with another patient, and because of the number of patients that come each day…the patients wait longer and we end up spending longer hours at work each day…*” – M1.

Data entry clerks had the role of transferring data from the forms on to the few computers available in the hospital. Due to the limited number of entry clerks at the hospital, this was a difficult task for them and data entry was a slow process with large backlogs. The records clerk that we interviewed described this as follows:

*“…we are not able to finish working on [data entry] before the end of the day, so it will pile up until the next day; and the next day, additional ones will be coming so…this makes the work even more difficult. Yes, so always, we do have a backlog …”* – C1.

The burdensome nature of the dual system had resulted in staff motivation and morale being low. It also resulted in more mistakes and less accurate data being entered into the system. Incomplete data also led to data having to be re-entered for subsequent patient contacts, further burdening the staff. One nurse’s sentiments summarizes this succinctly:

*“…since 2009 up to now we are still using this [hybrid system]… so gradually people have become fed up with it. They are tired and overworked and now the way people were excited to work with this new system has gone down... when you talk to them you can see that people are tired…it is too much”–*N1.

### Data capture process

Another challenge that was identified was in the data capture process. Ideally, a computerised MHIS allows data to be captured quickly and easily, and to be updated when needed [4, 19] [20]. However, this was not always the case with the new MHIS. One of the doctors described the cumbersome nature of the form that had to be filled:

*“...the idea is a good one but there are problems....the form is much too long, so when you are filling one for each patient, you end up spending almost twice as long with each patient... and you end up repeating a whole lot of information over and over again, which should not be necessary… Meanwhile you have other patients waiting to be seen...” –* D1.

A nurse also described how the filling of the form was exasperating:

“*…if a patient should come today… and medications are given to them…and maybe the patient is reacting to the drug in some way…that patient will have to come back to see the doctor, and each time the patient comes to see the doctor, we have to fill out a whole new form for them…*” – N2.

As a result of problems with initial data capture, the participants reported that their work was often disrupted and tended to become repetitive not only in transferring from paper to computer but also in updating and correcting computer files.

### Staff involvement

A further identified challenge was in staff involvement and training. Despite the care taken in setting up the new information system, some staff reported that they felt they had not been involved in the implementation process. All the clinical staff in our sample expressed this sentiment. Although they had been told that a new system was to be implemented, they believed their involvement in the process from the onset would have allowed some potential challenges to be forestalled.

*“…initially when the system came – that is, the new system – all the Records staff were happy, that now we were going to change the system of record keeping to a better one…our statistics and everything will be easier! But the people should have asked us how we want it, so that we can tell them what will make it easier...”* – C1.

Five of the participants also reported that they did not know what the data were for and therefore did not think it directly affected their work. Those staff members, who believed the MHIS would not assist their work directly, did not take care to fully implement the system. One nurse stated:

*“…honestly I don’t think it does us any good! It has just made our work harder! All that paper that is wasted, we don’t use it for anything! Well some of the Records people say that when they are doing their statistics, it is easier because everything is supposed to be online, but as for us, I’m not sure it has improved anything”* – N1.

### Resources and logistics

Properly designing the system and training staff who would use it are the first two steps in ensuring that the MHIS is properly implemented [3]. However, if logistic provision is not made to support the new system, there may still be challenges in running it properly. In developing the MHIS at the Accra Psychiatric Hospital, new hardware and software were made available. However, these proved to be inadequate to support the hospital’s information needs. As part of the implementation process, some computers were indeed provided for the MHIS data entry unit. However, there are close to 100 records generated each day which require entry onto the database [16]. The few computers are therefore not enough to facilitate this process. According to the records clerk:

*“…we have only three computers – it used to be less in the beginning – but now we have only three computers… [most days] we have 9 active [consulting rooms] – 9 of them! – and I know that one consulting room can see…close to 30 or 40 cases some days… So just imagine for all the 9 rooms, and look at the number of patients…at the end of the day, all the forms would be collected and sent to Records, then those working on the data entry – on just three machines – will sit down and begin the input…”* – C1.

In addition, factors such as erratic power supplies, wear and tear of the hardware, and maintenance needed to have been taken into consideration. One of the doctors empathized with the records clerks, given the resource limitations that they had to endure:

*“…apart from the computers, they also need constant power…with this so-called [energy crisis] it is even worse, almost every day the light goes off! And the generator does not always work properly you see…sometimes you can really see their frustration…”* – D2.

### Dissemination and use of data

A final challenge that was identified regards the dissemination of information obtained through the MHIS data. Information that is generated from the MHIS is ideally useful for, not only patients, service users and policymakers, but also for the healthcare staff. However, our participants perceived the system as a further layer of bureaucracy within the healthcare hierarchy of the hospital [16]. As one nurse lamented:

*“…I don’t know what they need it for; like the religion of the person, what do they need it for? Are they trying to see whether Christians have more psychiatric illness than Muslims? Or vice versa? Me, I don’t know why they will have to ask that information…”*

This may explain the apparent lack of interest in the optimal use of the MHIS by some of the participants – if it is simply extra work for which they did not receive feedback, then it is not surprising that they were not keen on exploring how to make the system function well for them.

Apart from the clinical level, there was also some dissatisfaction at the administrative level about the accuracy of the data that was generated through the system. The hospital administrator expressed his frustration at the data that he periodically received:

*“...they [the records staff] were the ones we were expecting to do better... instead, that is where most of the garbage is coming from... they often provide us with data which are not realistic... Considering the backlog of unentered data...when you ask for reports, they give you data as though it is real time data and when you question further, you realize that they estimated the information to get those results and it is usually based on their manual data...or so they say” –* A1.

This could perhaps explain why reports were not being fed back to the users as often as was expected. It also speaks to the difference in expectations for data use between the clinical staff, the clerks, and the administration of the hospital.

Ideally, in addition to capturing patient information, an MHIS should also coordinate the behaviour of staff and improve decision-making processes; however, this can only be achieved when information is collected properly, and when this collated data and the work processes involved in collecting that data trickle down to the users at all levels of the organizational structure.

## Discussion

In this paper, we sought to examine the challenges that staff at the Accra Psychiatric Hospital had encountered with the newly implemented MHIS. Generally, the participants all agreed that the old paper-based system was not ideal, and agreed that a new computerized system was needed. The primary reason cited for this need was to ease record keeping workload. None of our participants was aware that the new system could have benefits for clinical work such as fewer prescription errors, increased adherence to treatment guidelines and improved clinician communication, as some literature has reported [21, 22]. This perception prevailed despite the information sessions that were reportedly held during the new system’s implementation. There is therefore little engagement with the new MHIS among the clinical staff of the hospital as they report they have not found it useful.

Although the administration generates periodic reports from data collected by the system, our clinical participants reported no knowledge of such reports. This could perhaps explain the apparent lack of interest in the function of the MHIS by the participants – if it is simply a routine (and added) work process for which they did not receive feedback, then it should not be surprising that there is not much vested interest in making it work optimally.

Staff at the hospital appeared to be going through the motions of ‘using’ the new MHIS without actually believing in its usefulness. This differed slightly from what the literature suggests, where studies in other countries reported that health workers generally agreed that a properly structured MHIS resulted in better clinical communication and therefore showed a willingness to work with an improved system [6, 23]. In the case of our study setting, the health workers did not appear to believe so, but this may be a reflection of the flaws in the current system.

The lack of enthusiasm for working with the new MHIS seems to stem largely from the manner in which it was introduced. None of the clinical participants recalled being told during their training that it was to be useful for adherence, for improved clinician communication and for patient management – all of them reported being told the MHIS was to improve recordkeeping at the hospital and indeed, they believed it would do so if it were made fully computerized. All the participants therefore agreed that the extra workload was not necessarily a result of the system per se, but rather a result of the current semi-computerized nature of the current system.

However, apart from the manner in which it was introduced, it does appear that the current system’s design does not allow the MHIS to meet the needs of all the different categories of its users. Given that the MHIS was envisioned to meet both clinical and administrative needs of the hospital, the limited usability of the system may be a reflection of flaws at deeper levels of work at the hospital. Although the implementation report suggested that various stakeholders were involved in its design, there appear to be differences in actual needs and what the MHIS is currently providing.

The increased workload that had resulted from the cumbersome nature of the form also presents a different set of implications. This is because ideally, an electronic system should be such that information that is entered can be easily retrieved and updated or modified [4, 20, 24]. It is therefore quite ironic that the participants described the new system as rather increasing their workload. Since our study ended, work has begun to develop shorter forms as well as separate forms for returning patients, in a bid to strengthen the system. A recent article by some authors involved in the initial set-up, also reported that a shorter form has been designed and is being used [25].

Despite the reported shortfalls of the new system and the attendant increase in workload, the workers should not be able to simply choose to not use the forms without consequences. Yet, from observations and admission by some participants, some of them simply ignore the form. This does not bode well for the hospital and indeed, may affect the intended benefits of the MHIS. If this is to be corrected, the management of the hospital need to take a more proactive role in ensuring adherence to the new system. It was not clear from the data to what extent managers empathized with the frustrations of their staff, or how much they valued the outcomes envisioned with the new MHIS. However, in order for the clinical staff’s use of the system to function appropriately, strong input from the management would also be needed.

In addition to staff attitudes, for the MHIS to function optimally, resources are required from the outset. These resources include provision for training of staff who would collect the data, enter the data, as well as those who would monitor and maintain the system. From the report of the MHaPP project, this training may have been done; however, it does not appear to have done what was intended, as our participants reported being unaware of much of the other functions of the MHIS beyond record keeping. This suggests that there is perhaps the need for better-structured information sessions, or at the very least, the need for periodic training updates to ensure that all levels of the system are in tune.

Finally, additional resources that are needed include sufficient computer hardware and software, as well as other logistical support. Although two computers were provided by the MHaPP, and additional ones have been bought by the hospital since then, the amount of data that is generated through the doctors’ offices requires more computers to be allocated to data entry, and a reliable power supply to avoid interruptions.

### Limitations of the study

Although important findings were obtained from this study, there were some limitations, which are important to highlight. The primary limitation was at the data collection stage. As has been discussed above, a number of staff at the hospital were reluctant to participate in this study. Apart from some doctors who cited busy schedules as their reasons, other clinical staff were cautious and sometimes unwilling to speak to someone whom they considered an outsider. The reason they cited for this suspicion was past experience with journalists and researchers whose only objective (in their opinion) was to paint them in a bad light. As a result of this reluctance, as well as time constraints, the data could only be collected from a limited number of workers. As such, the views and opinions expressed may not necessarily reflect the larger population of the hospital workforce. Their reluctance may also have influenced their responses to our questions.

A further limitation was the fact that data was collected from only one of the three hospitals where the MHIS was implemented. Obtaining data from the other hospitals would provide a more representative picture of the state of the MHIS. It may also have allowed comparison of work processes between the hospitals to identify potential strengths and weaknesses. The problem of data saturation would also have been mitigated if the other hospitals had formed part of our study. However, due to time and logistic constraints, it was not possible to collect data at the other hospitals at the time of data collection.

## Conclusions

As important as it is to have a mental health information system, whether or not a modern MHIS works in any country – and particularly LMICs – is largely dependent on its being properly implemented and maintained. Based on the challenges identified in our evaluation of the MHIS at the Accra Psychiatric Hospital, setting up a new system does not guarantee its success. There are various factors which need to be properly considered in order for the MHIS to be successful and useful. However, these challenges do not apply only at an institutional level. Further success can be obtained when the place of mental health in district, regional and national health discourse improves. Governments and policymakers must be willing to invest in supporting systems which will lead to scaling up mental health services and access to care in their countries. Such national involvement can allow the MHIS to function optimally – by collecting, processing and disseminating data appropriately, enhancing clinician communication, and reducing errors.

However, this can only be achieved when changes are made at all levels of the healthcare hierarchy. The ongoing involvement of policymakers, medical staff, technicians and administrators in the implementation process may help ensure that the MHIS functions usefully. Involving all stakeholders in this process can also help to ensure better compliance. In addition, when there is agreement and understanding of the benefits of the new system, there may be full engagement in making the MHIS function properly. In this way, the MHIS data can be reliably used in informing policy change and advocacy for improved services. At this stage however, it is important to anticipate and plan for possible implementation and sustainability challenges.

## Abbreviations

IPA: Interpretative phenomenological analysis
LMIC: Low and middle-income country
MHaPP: Mental Health and Poverty Project
MHIS: Mental health information system
